# Men Wanted

**Published:** 1856-12

**Authors:** 


### HALL'S JOURNAL OF HEALTH.

OUR LEGITIMATE SCOPE IS ALMOST BOUNDLESS: FOR WHATEVER BEGETS PLEASURABLE AND HARMLESS FEELINGS, PROMOTES HEALTH; AND WHATEVER INDUCES DISAGREEABLE SENSATIONS, ENGENDERS DISEASE.

VOL. III.] DECEMBER, 1856. [NO. XII.

### MEN WANTED.

Passing through the Bowery some time ago, a flag was observed, extending over the side-walk, in large black letters, "MEN WANTED."

So they are; wanted everywhere; for the pulpit, for the press, for the judge's bench and the halls of legislation. In our opinion, it requires a man to do anything well, to black a shoe, construct a locomotive or build a ship; men of mind and men of body; stout men, strong men, men of vigor and of high health are "wanted."

But the object for which "men" were "wanted" at the place above, was enlistment as soldiers for the United States Army. All over the world the best specimens of physical humanity are selected for the army. The halt, the lame, the deaf, the blind, the little, the sickly, the deformed, are turned off with contempt by the inspecting officer. Of such importance is this physical perfection in some despotic countries, that boys are purposely trained by all sorts of gymnastic exercises, until their feats of agility are scarcely surpassed by the professed circuit rider or rope dancer.

So it seems that tyrants and despots are the first to perceive the value of physical training for developing the highest capabilities of man.

But if it is thought of such importance to have well-developed men for purposes of killing their fellows, it might be well to inquire if it would not be advantageous to train men to high bodily health and physical perfection for better callings and for nobler purposes. How long will it take to teach the world, that physical perfection and mental power of the highest order go hand in hand. We cannot say that the world is any the better for the *whole work*, of any mind that operated through a sickly body.

